# Reference markers of bone turnover for prediction of fracture: a meta-analysis

**DOI:** 10.1186/s13018-019-1100-6

**Published:** 2019-02-28

**Authors:** Aixian Tian, Jianxiong Ma, Kaiqiang Feng, Zhaojie Liu, Lei Chen, Haobo Jia, Xinlong Ma

**Affiliations:** 1 Tianjin Hospital, Tianjin University, Tianjin, 300211 China; 2 Department of Clinical Laboratory, Tianjin Hospital, Tianjin, 300211 China; 3 Department of Otolaryngology, The Second Hospital of Tianjin Medical University, Tianjin, 300211 China; 4 Department of Orthopedics Institute, Tianjin Hospital, Tianjin, 300211 China

**Keywords:** BTMs, PINP, CTX, Fracture

## Abstract

**Objective:**

To explore whether bone turnover biomarkers (BTMs), i.e., C-terminal telopeptide of type I collagen (CTX) and procollagen type I aminoterminal propeptide (PINP), are associated with fracture.

**Methods:**

We searched electronic database including PubMed, Embase and Cochrane Library, and the reference lists of relevant articles published from inception to August 22, 2018. An updated meta-analysis was performed to assess the prediction value of CTX and PINP in fracture.

**Results:**

Nine articles met our inclusion criteria and were included in the meta-analysis. The crude and adjusted effect size between PINP and fracture were extracted from two and five studies, respectively. PINP was not associated with fracture incidence without adjusting covariates (crude GR, 1.03; 95% CI, 0.91–1.17). After adjusting for potential confounders, PINP demonstrated a significant positive association with fracture (adjusted GR, 1.28; 95% CI, 1.15–1.42). In the subgroup analysis of studies after adjusting covariates, there were significant associations in women. Both the crude (1.16, 95%CI, 1.04–1.20) and adjusted GR (1.20, 95%CI, 1.05–1.37) shown positive relationships between CTX and fracture, which were extracted from four and six studies, separately. The sensitivity analysis confirmed the stability of the results. In the subgroup analysis of studies after adjusting covariates, there were significant associations in the subgroups of elderly, female, and hip fracture patients.

**Conclusions:**

Our results indicate a statistically significant but modest association between BTMs (s-PINP or s-CTX) and future fracture risk after adjusting for BMD and clinical risk factors. The causal relationship between the two clinical conditions requires future validation with more standardized studies.

**Registration number:**

CRD42018107879

**Electronic supplementary material:**

The online version of this article (10.1186/s13018-019-1100-6) contains supplementary material, which is available to authorized users.

## Background

Fracture is a worldwide public health problem because of the increased morbidity, mortality, and financial costs [1]. However, the ability to predict and prevent fractures is limited. The current approaches for predicting who might fracture are largely based on the measurement of bone mineral density (BMD) and the inclusion in risk calculators of certain clinical risk factors. Such risk calculators include FRAX, QFRACTURE, and the Garvan calculator [2]. However, BMD indicated osteoporosis only in 30–50% of patients with major fragility fracture [3]. And the prognostic value of clinical risk factors alone in FRAX is comparable to that of BMD alone [4]. There is an imperious need of identifying additional fracture risk factors not included in currently available strategies.

Bone turnover biomarkers (BTMs) reflect bone formation and resorption and therefore inform the status of bone remodeling. Attractive features of these markers are that samples of blood are easily collected, a variety of assays is available, and sample collection is relatively noninvasive. The development of markers of BTMs has provided an important tool in the clinical and preclinical assessment of bone active interventions. A working group of the International Osteoporosis Foundation (IOF)/International Federation of Clinical Chemistry (IFCC) and Laboratory Medicine Bone Markers Working Group identified one bone resorption marker, C-terminal telopeptide of type I collagen (CTX), and one bone formation marker, procollagen type I aminoterminal propeptide (PINP), as the most promising bone turnover markers [5]. Serum PINP (s-PINP) is generated during the synthesis of type I collagen, and serum CTX (s-CTX) is a product of the breakdown of type I collagen containing pyridinium crosslinks.

Recently, there are numerous of studies tried to examine bone turnover marker levels in relation to fragility or osteoporotic fractures. Of those studies, some studies found a positive relationship between bone turnovers and the incidence of fracture [6, 7]. However, some recent researches represented different results [8–10]. A meta-analysis published in 2014 has been conducted for serum CTX-1 and PINP in ten prospective cohort studies of untreated participants [11]. And it turned out that for 1 standard deviation (SD) increase, the risk of all fractures was 1.18 with CTX and 1.23 with PINP. However, these results were not adjusted for BMD and/or other potential confounders. And the performance characteristics of clinical risk factors (CRFs) and BMD were proved to be strongly associated with the prediction value of fracture risk. For example, in a large meta-analysis, CRFs alone predicted hip fracture with a GR (the gradient of risk) of 2.1/SD and the use of BMD alone provided a higher GR (3.7/SD), and this was improved further with the combined use of CRFs and BMD (4.2/SD) [12].

To our knowledge, data should also be adjusted for bone mineral density and clinical risk factors so that the BTM are evaluated for their value in fracture risk prediction algorithms. Therefore, based on existing evidence, a study needs to be updated and critically evaluates the current evidence-based information about the prediction of fracture by BTMs. In our study, a comprehensive meta-analysis was conducted to assess the potential value of serum PINP or CTX in fracture risk prediction.

## Methods

### Searches

Two reviewers independently searched electronic database including PubMed, Embase, and Cochrane Library based on logic combination of keywords and text words from inception to August 22, 2018, and updated them on October 14, 2018. Search terms included “procollagen Type I N-terminal peptide”, “ PINP”, “collagen type I trimeric cross-linked peptide”, “ CTX”, and “fractures”. Search terms were combined using the Boolean operators “AND” or “OR”. The search was restricted to studies of human participants, but we set no search restrictions on follow-up time, patients’ age, study size, and the language of articles. Reference lists of relevant articles were manually searched to identify additional trials.

### Study inclusion and exclusion criteria

Studies eligible for inclusion were prospective cohort studies of s-PINP or s-CTX measured at baseline in untreated individuals. Nested case–control and case–cohort studies were also allowed. The primary outcome was the first incident fracture in middle-aged or older men and women. We excluded cross-sectional studies and articles that test OINP or CTX in the urine. Basic science studies, reviews, editorials, letters, case reports, and studies without comparison groups were also excluded.

### Data extraction

Two members of the study team independently assessed all titles and abstracts of identified reports for eligibility. We obtained the full text if at least one of the reviewers judged a study to be eligible. Disagreements on inclusion were resolved by consensus. For each study, patients’ characteristics including mean age, sex, duration of follow-up, state of fasting, site of fracture, trial size, and results were individually extracted.

### Study quality assessment

The quality of the included citations, which was assessed by the Newcastle-Ottawa Scale, was independently scored by two authors. The scale was widely used for the evaluation of non-randomized controlled trials with respect to selection, comparability, and outcome/exposure of the enrolled studies [13]. The highest score was 9 points, and a score of ≥ 7 points was suggestive of a high-quality study. Discrepancies between both reviewers were resolved by discussion or evaluated by the corresponding author.

### Outcome measures

The primary outcome of interest was the crude and adjusted associations of BTMs (i.e., s-PINP or s-CTX) with incidence of fracture, expressed by HR for fracture per SD difference (the GR) and 95% confidence interval (CI). It is hard to combine studies in an analysis. This is because the included studies reported the results in various ways. Some define high bone turnover as being more than 1 or 2 SDs above the mean or in the highest tertile, quartile, or quintile. To merge the results, a uniform metric was needed. The metric which we selected was the GR. Besides, if the results were reported in more than one way, the GR was extracted preferentially, and if the GR was absent, we used the HR per unit of measurement. Where neither was available, the ratio of quartiles was extracted and transformed into GR by using a mathematical approximation as previously described [11, 14].

### Data synthesis and statistical analysis

All results summarized using the Stata software package (version 12.0). We calculated GR with 95% CI. Data were pooled using a random effects model to give a more conservative estimate of the effect. Heterogeneity between studies was assessed using both the *I*^2^ statistic with a cut off of ≥ 50%, and the χ^2^ test with a *P* value < 0.10 used to define a significant degree of heterogeneity [15]. The subgroup analysis was conducted based on gender (male or female), age (more than 65), and site of fracture (hip fracture). In order to assess the influence of individual studies on the pooled result, we conducted a sensitivity analysis by excluding each study one by one and recalculating the combined estimates on the remaining studies.

The funnel plot along with Begg’s test was performed to plot the log GR against its standard error for evaluation of publication bias, while the extent of asymmetry was assessed by Egger’s unweighted regression asymmetry test [16]. We used two-tailed *P* values and *P* < 0.05 was regarded as statistical significance except that for determining heterogeneity (*P* < 0.1)

## Results

### Study characteristics and quality assessment

**Fig. 1:**
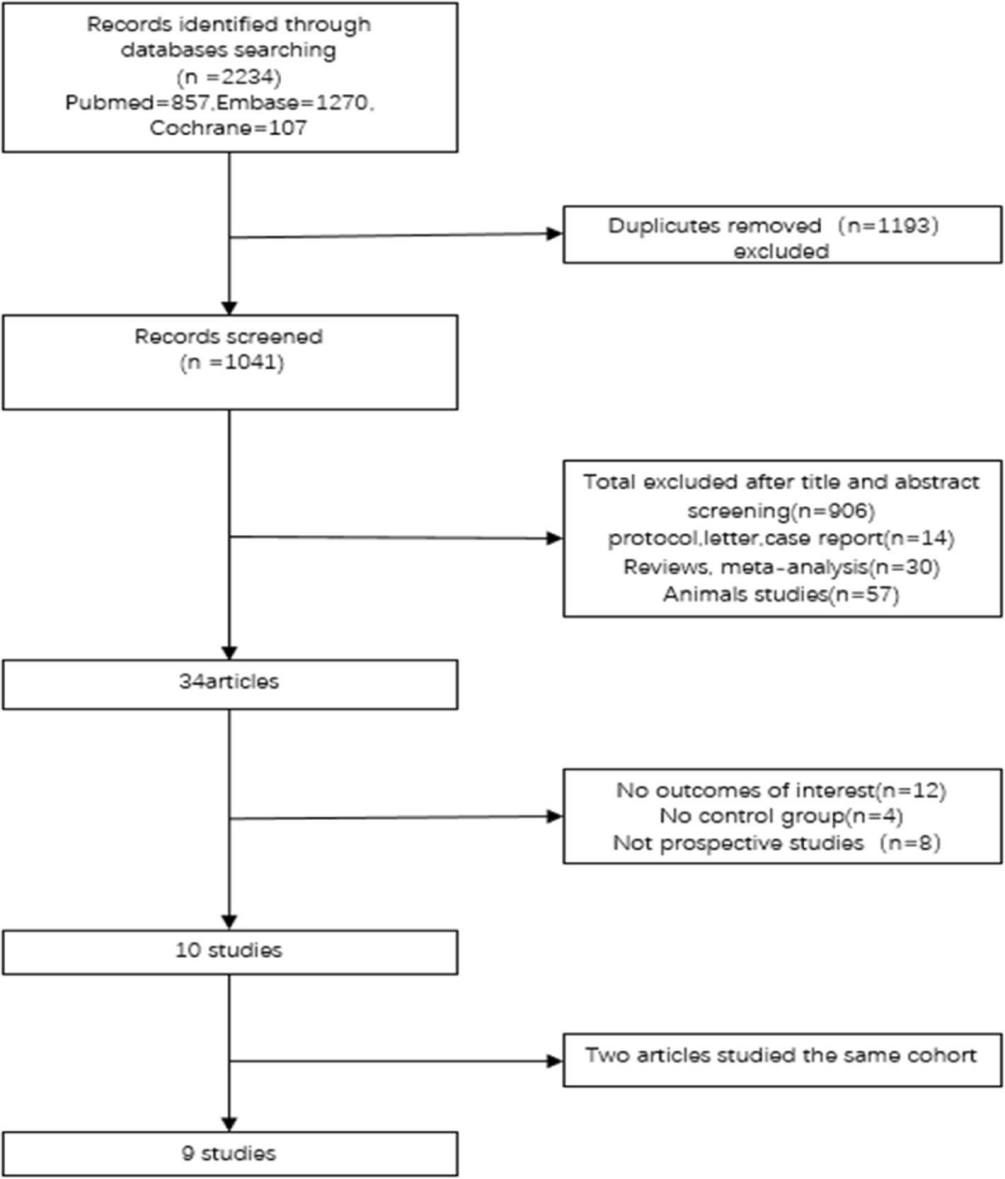
PRISMA flow diagram for the study selection process

A flowchart of study search and selection was presented in Fig. 1. In brief, we identified 1041 references in our literature search and out of 34 potentially eligible studies, 10 articles describing 9 trials met our inclusion criteria and were included in the meta-analysis [6–8, 10, 14, 17–21]. Gerdhem et al. [17] and Ivaska et al. [18] studied the same cohort but had different follow-up time. The study with the shorter follow-up was selected for inclusion in the meta-analysis because the study of Ivaska et al. [18] reported a time interaction. Finally, nine articles were chosen in our study [6–8, 10, 14, 17, 19–21].

Table 1 lists the characteristics of eligible and included studies. A total of 11,572 participants were included in this meta-analysis. The average follow-up time ranged from 2.0 to 7.13 years. In terms of the targeted population, the age of the observed population ranged from 45 to 89 years. Five studies enrolled elderly people only (> 65 years of age) [7, 17, 19–21], and other four studies enrolled both elders and middle-aged persons [6, 8, 10, 14]. Six studies presented results for women [7, 8, 10, 14, 17, 20], two studies presented results for men [19, 21], and one article presented results for men and women combined [6]. Regarding the fasting state of included participants, five studies tested participants in fasting state [6, 8, 10, 14, 21], three studies were in non-fasting state [17, 19, 20], and one study was not clear about the fasting state [7]. In terms of fracture site, six studies reported hip fracture [6–8, 17, 20, 21], other studies presented various sites of fracture.

**Table 1:** Characteristics of the included studies

Study	BTM	Follow-up (years)	Sex	Age (years)	Number of participants	Population/setting	Fasting
Chapurlat [7]	s-CTX	3.3	F	> 75	854	Population-based registers	–
Garnero [14]	s-CTX	5	F	50–89	435	Healthy untreated postmenopausal	Yes
Gerdhem [17]	s-CTX	6.5	F	75	1040	Population-based nursing home	No
Meier [19]	s-PINP,s-CTX	6.3	M	> 70	151	All in city	No
Dobnig [20]	s-CTX	2	F	> 70	1664	Nursing home	No
Bauer [21]	s-PINP,s-CTX	4.6	M	> 65	5995	Advert and mass mailing	Yes
Shigdel [10]	s-PINP,s-CTX	6.6	F	> 50	433	Population-based registers	Yes
Dai [6]	s-PINP,s-CTX	5	F and M	45–74	200	Population-based cohort	Yes
Crandall [8]	s-PINP,s-CTX	7.13	F	50–79	800	Clinical centers	Yes

The study quality scores are summarized in Table 2. The range of quality scores was from 4 to 8; the median score was 7. High-quality studies (i.e., those having ≥ 7 awarded stars) included six studies [6–8, 10, 17, 19].

**Table 2:** Quality assessment by using the Newcastle-Ottawa Scale for the included studies

	Chapurlat [7]	Garnero [14]	Gerdhem [17]	Meier [19]	Dobnig [20]	Bauer [21]	Shigdel [10]	Dai [6]	Crandall [8]
Cohort study									
Representativeness of the exposed cohort	+	−	+	+	−	+	+	+	+
Selection of the unexposed cohort	+	−	+	+	+	+	+	+	+
Ascertainment of exposure	+	+	+	+	+	+	+	+	+
Outcome of interest not present at the start of the study	−	−	−	−	−	−	−	−	−
Control for important factor or additional factor									
Study controls for age/sex	+	−	+	+	+	−	+	+	+
Study controls for any other confounding factors	−	−	+	−	+	−	+	+	−
Outcome assessment	+	+	+	+	+	+	+	+	+
Follow-up long enough for outcomes to occur	+	+	+	+	−	+	+	+	+
Adequacy of follow-up of cohorts	+	+	+	+	+	+	+	+	+
Total quality scores	7	4	8	7	6	6	8	8	7

### Overall analysis

**Fig. 2:**
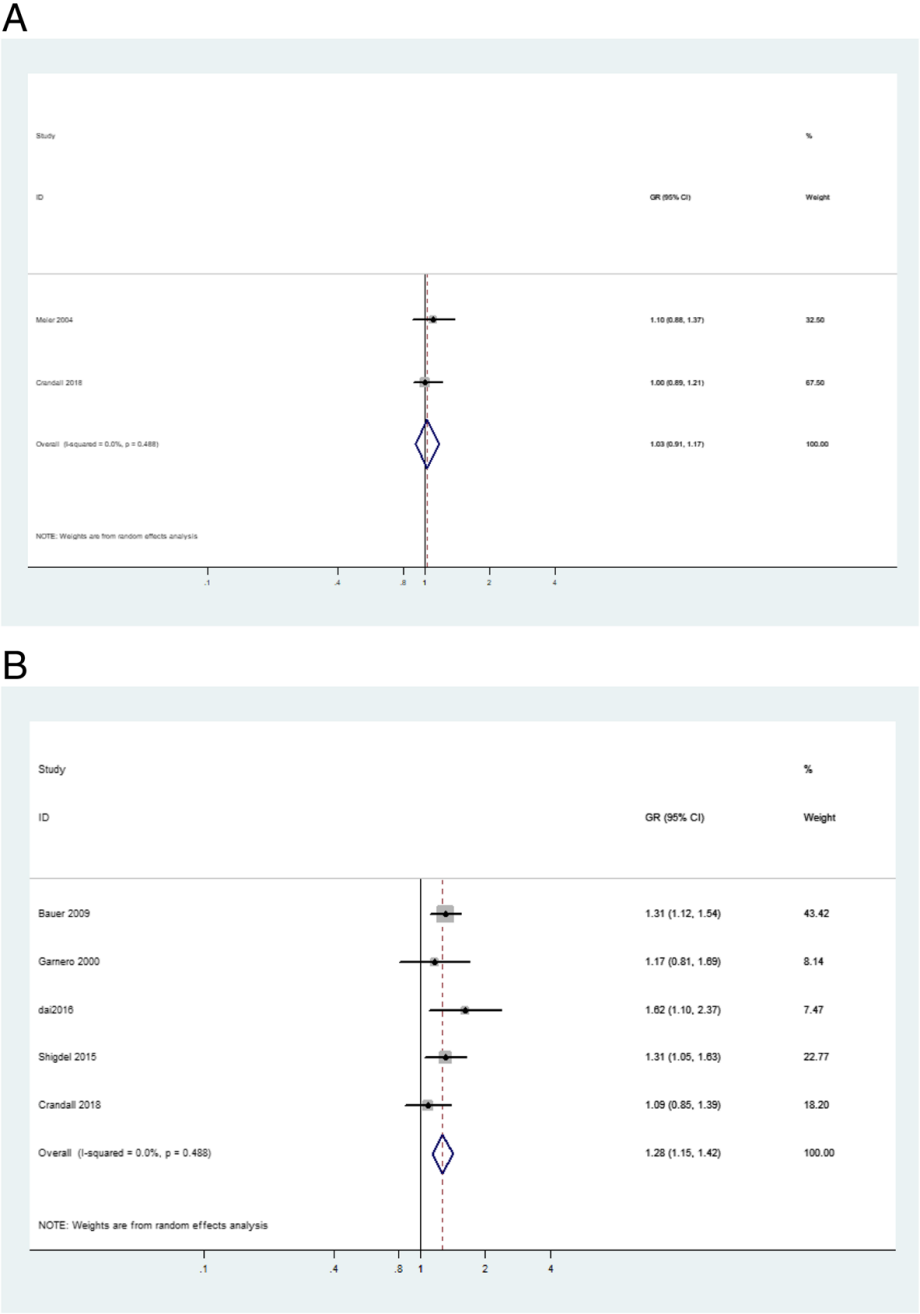
Forest plot of the **a** crude and **b** adjusted associations between PINP and fracture

The crude GR and adjusted GR between s-PINP and fracture was extracted from two studies [8, 19] and five studies [6, 8, 10, 14, 21], respectively (Table 2). As shown in Fig. 2, s-PINP was not associated with fracture without adjusting for covariates (crude GR, 1.03; 95% CI, 0.91–1.17). After adjusting for potential confounders such as age, body mass index, mobility score, past fractures, and hip BMD, s-PINP demonstrated a significant positive association with fracture (adjusted GR, 1.28; 95% CI, 1.15–1.42), which indicated that a 1 SD increment in s-PINP was associated with an increased risk of fracture of 28%. The subgroup analysis showed that s-PINP tended to be associated with fracture in women (adjusted GR, 1.25; 95% CI, 1.08–1.45). It turned out that s-PINP was not associated with hip fracture (adjusted GR, 1.29; 95% CI, 0.88–1.90). The results of the Egger’s and Begg’s test indicated the absence of publication bias in adjusted GR (*P* values = 0.923) (Additional file 1: Figure S1).

**Fig. 3:**
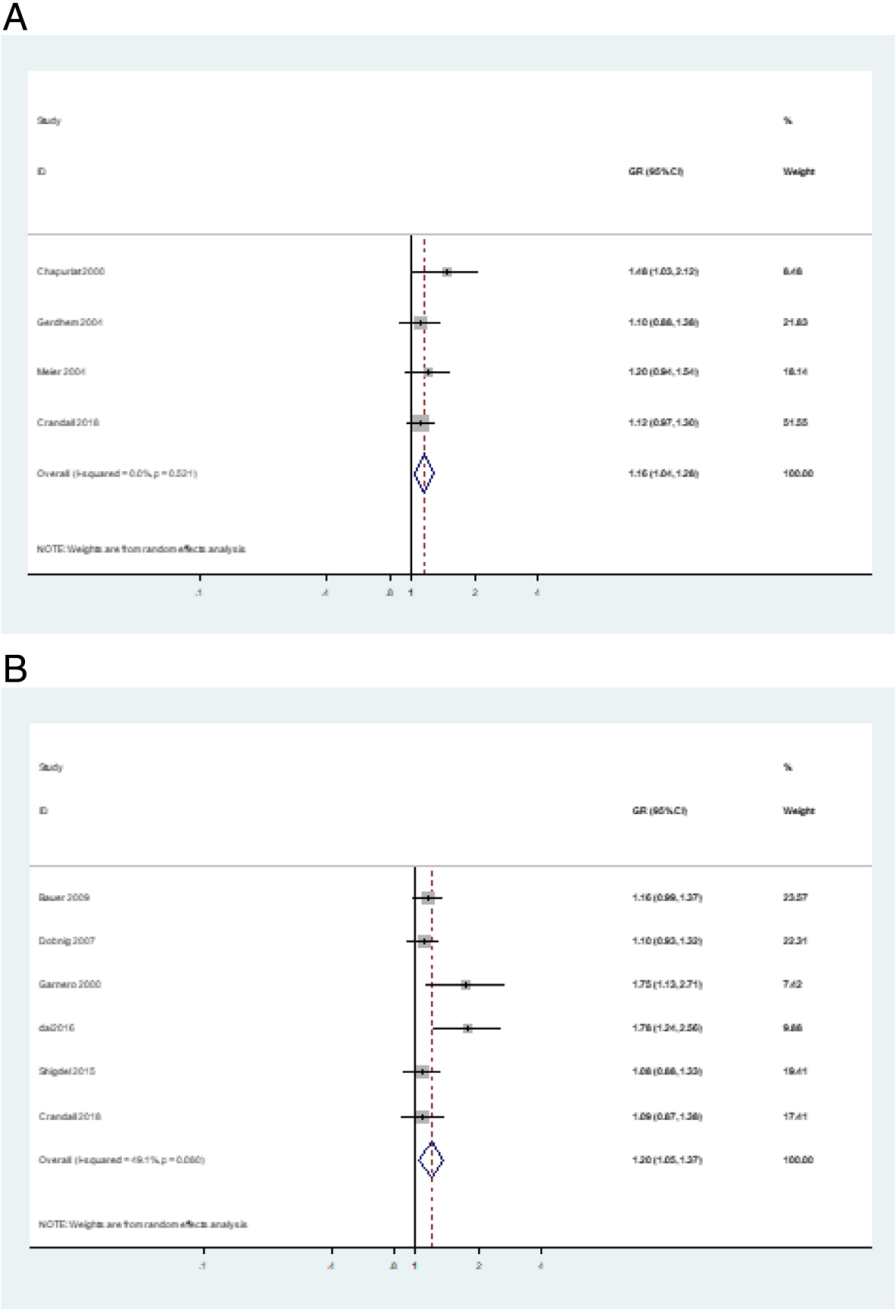
Forest plot of the **a** crude and **b** adjusted associations between CTX and fracture

The crude GR and adjusted GR between s-CTX and fracture was extracted from four studies [7, 8, 17, 19] and six studies [6, 8, 10, 14, 20, 21], separately (Table 3). Both crude GR (1.16, 95%CI, 1.04–1.20) and adjusted GR (1.20, 95%CI, 1.05–1.37) shown a significant positive result. And subgroup analysis showed a mildly significant association between s-PINP and fracture in the group of elderly, female, and hip fracture patients (Fig. 3). The results of the Egger’s and Begg’s test indicated the existence of publication bias in adjusted GR but not in crude GR (*P* values = 0.034 for crude GR and 0.178 for adjusted GR) (Additional file 1: Figure S1).

**Table 3:** The relationship between s-PINP and fracture risk

Study	Fracture outcome	Type of unit	Unadjusted HR or OR (95% CI)	Adjusted HR or OR	Covariates
Garnero [14]	All	Highest quartile vs. three lower quartiles		1.3 (0.7–2.4)	Age, presence of prevalent fractures, and physical activity
Meier [19]	All	Highest vs lowest quartiles	1.4 (0.8–1.6)		
	All	Per SD	1.1 (0.9–1.4)		
Bauer [21]	Hip	Highest quartile vs. three lower quartiles		2.13 (1.23–3.68)	Age and clinic
	Nonvertebral	Highest quartile vs. three lower quartiles		1.57 (1.21–2.05)	
	Hip	Highest quartile vs. three lower quartiles		1.16 (0.57–2.36)	Age, BMI, race, diabetes, grip strength, clinic, and baseline total hip BMD
	Nonvertebral	Highest quartile vs. three lower quartiles		1.31 (0.98–1.74)	
Shigdel [10]	Hip, wrist humeral	Per SD		1.31 (1.05–1.63)	Age, height, weight, and femoral neck areal bone mineral density
Dai [6]	Hip	Highest vs lowest quartiles		6.63 (2.02–21.81)	Age, sex, dialect group, date of study enrollment, and date of biospecimen collection, BMI, level of education, smoking status, physical activity, soy isoflavones, β-carotene, diabetes mellitus
		Per SD		1.62 (1.10–2.37)	
Crandall [8]	Hip	Highest vs lowest quartiles	1.09 (0.73, 1.63)	1.24 (0.65, 2.35)	Body mass index, years of education, whether living with a partner, parity, smoking, fall history in past year, history of previous fracture, family history of hip fracture, past use of menopausal hormone therapy, and vitamin D intake

### Sensitivity analysis

**Fig. 4:**
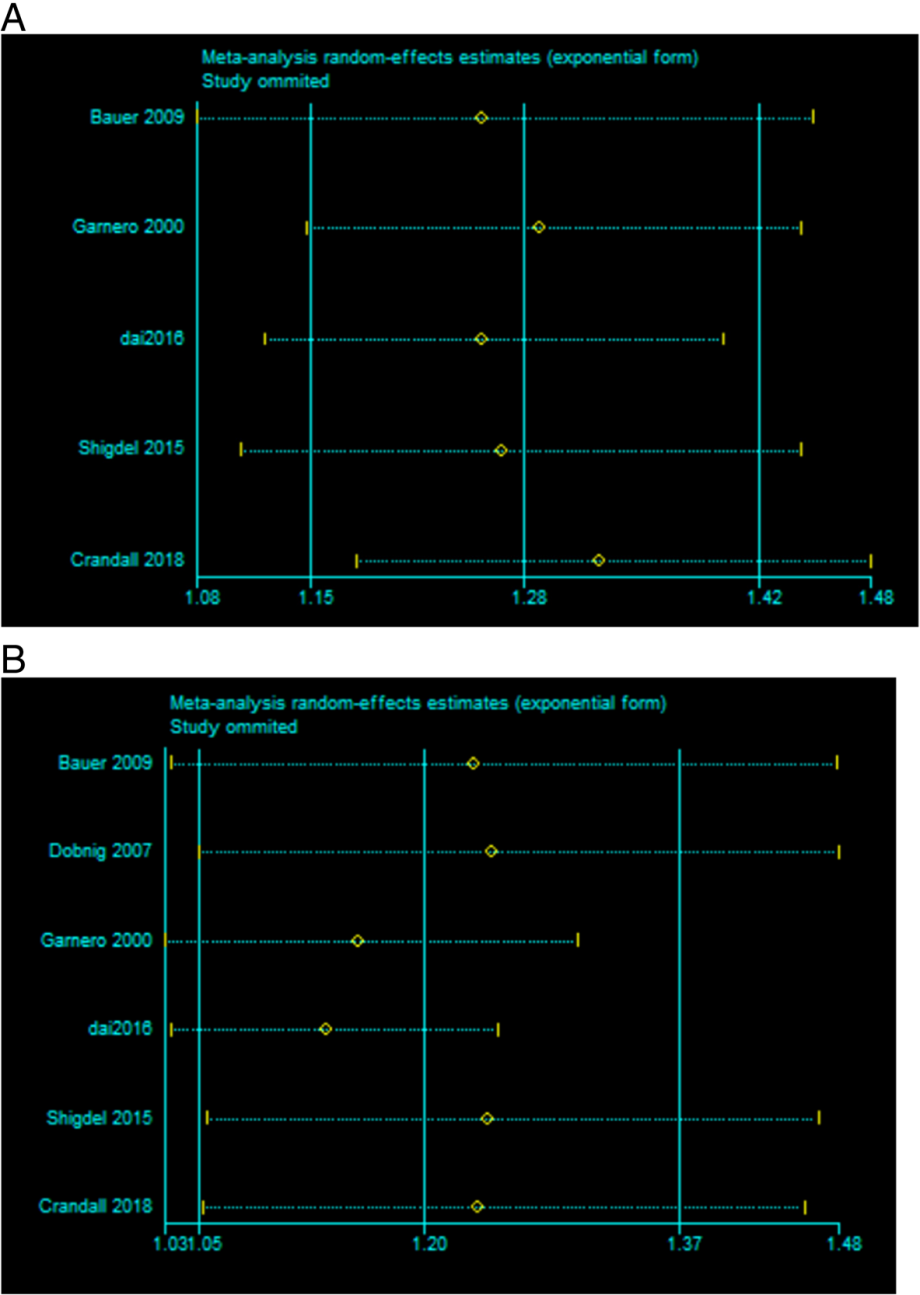
Sensitivity analysis of adjusted GR: **a** PINP, **b** CTX

In sensitivity analyses, we recalculated the combined results by excluding one study per iteration. The adjusted GRs between s-PINP and fracture ranged from a low of 1.25 (95% CI: 1.08, 1.45) to a high of 1.32 (95% CI: 1.18, 1.48) via omission of the study by Bauer et al. [21] and via omission of the study by Crandall et al. [8] respectively. And the adjusted GRs between s-CTX and fracture ranged from a low of 1.14 (95% CI: 1.03, 1.25) to a high of 1.25 (95% CI: 1.05, 1.48) via omission of the study by Dai et al. [6] and via omission of the study by Dobnig et al. [20] respectively. Both results were similar without great fluctuate (Fig. 4).

## Discussion

The present study aimed to integrate the most updated evidence to evaluate the association between BTMs (i.e., s-PINP or s-CTX) and the incidence of fracture. The overall results indicated that s-PINP and s-CTX were positively associated with fracture after adjusting for relevant covariates when we chose the expression of risk as the gradient of fracture risk per SD difference in BTM. This combined estimate was robust across sensitivity analyses. The stratified analysis further showed that there was a statistically significant but modest association between CTX and fracture in the group of elderly, female, and hip fracture patients. Besides, there was a significant association between PINP and fracture in women.

The ability to predict fractures is limited and needs further development. BTMs reflect bone formation and resorption and therefore inform the status of bone remodeling. Thus, the measurement of BTMs may serve either as an independent diagnostic and prognostic index or as a complementary indicator to BMD for fractures. It was suggested that PINP and CTX-I were the best risk predictors for fractures [2, 6]. A number of studies have attempted to make certain the impact of PINP and CTX-I on incident fracture in the middle-aged and elderly people, but failed to reach an agreement [2]. To our best of knowledge, data should also be adjusted for BMD and clinical risk factors so that the BTM are evaluated for their value in fracture risk prediction algorithms. Collectively, based on the conflicting results across studies and the significance of both clinical conditions, a meta-analysis was conducted to investigate the independent role of BTM in fracture risk prediction.

In line with the recommended analytes by the IOF and IFCC, our data confirmed that PINP and CTX-I could be the risk predictors for fractures because BTMs play a central role directly and indirectly in the mechanical resistance of the skeleton [22]. Besides, our main results were consistent with a previous meta-analysis showing there was a moderate but significant association between s-PINP, s-CTX, and risk of fracture, which was not adjusted for BMD and/or other relevant covariates [11]. As for the crude GR between s-PINP and fracture, there were just two articles included in our study, so it may need more evidence to illustrate the relationship.

It is not surprising that BTMs are significantly associated with fracture incidence in women more than 45 years old. At menopause, there is an acceleration in the rate of bone loss, and this is naturally related to the increase in bone turnover. And higher levels of BTMs were associated with higher cortical porosity and thinner cortices, which may lead to the incidence of fracture [10]. As we know, the elderly have always been subject to fractures. BTMs increased during aging in both men and women and have been suggested to be independent risk factors for fractures [23, 24]. Therefore, there might be age interaction between the BTMs and fracture risk.

Other forms of utilization about BTMs should also be taken into consideration in the future study. Joint effect of serum PINP and CTX-I on the risk of hip fracture seems to be stronger [6]. Lower serum PINP/CTX ratio demonstrated an inverse dose–effect relationship with the prevalence of nonvertebral fractures [9] (Table 4).

**Table 4:** The relationship between s-CTX and fracture risk

Study	Fracture outcome	Type of unit	Unadjusted HR or OR (95% CI)	Adjusted HR or OR	Covariates
Chapurlat [7]	Hip	Highest quartile vs control	1.9 (1.05–3.4)		
Garnero [14]	All	Highest vs lowest quartiles		2.1 (1.2–3.8)	Age, presence of prevalent fractures, and physical activity
Gerdhem [17]	All	Highest quartile vs. three lower quartiles	1.18 (0.81–1.70)		
	Hip		1.01 (0.48–2.11)		
	Vertebral		1.94 (1.05–3.58)	1.58 (0.83–2.98)	Lumbar spine BMD
Meier [19]	All	Highest vs lowest quartiles	1.6 (0.8–3.3)		
	All	Per SD	1.2 (0.98–1.6)		
Dobnig [20]	Hip	Per increment of 1 ng/mL		1.27 (0.45–3.6)	Age, BMI, mobility score, past fractures, creatinine clearance rate, calcaneal stiffness
	Nonvertebral			1.41 (0.77–2.6)	
Bauer [21]	Hip	Highest quartile vs three lower quartiles		1.76 (1.04–2.98)	Age and clinic
	Nonvertebral			1.29 (0.99–1.69)	
	Hip			1.04 (0.55–1.97)	Age, BMI, race, diabetes, grip strength, clinic, and baseline total hip BMD
	Nonvertebral			1.07 (0.80–1.42)	
Ivaska [18]	All	Per SD	1.13 (1.01,1.27)		
	Vertebral		1.32 (1.05,1.67)		
Shigdel [10]	Hip, wrist	Per SD		1.08 (0.88–1.33)	Age, height, weight, and femoral neck areal bone mineral density
Dai [6]	Hip	Highest vs lowest quartiles		4.92 (1.67–14.51)	Age, sex, dialect group, date of study enrollment, BMI, level of education, smoking status, physical activity, diabetes mellitus
		Per SD		1.78 (1.24–2.56)	
Crandall [8]	Hip	Highest vs lowest quartiles	1.33 (0.91, 1.96)	1.25 (0.68, 2.30)	Body mass index, years of education, whether living with a partner, parity, smoking, fall history in past year, history of previous fracture, family history of hip fracture, past use of menopausal hormone therapy, and vitamin D intake

There are several limitations in this meta-analysis which need to be considered. Firstly, in the absence of access to primary data, we standardize the metric of predictive power (the GR) to maximize the use that can be made of publications that used differing indices of risk. Secondly, although most studies report a positive association between BTM and fracture risk, each study has several fracture endpoints, so these could be false positives. It would be better to define the reference range of BTM along with single fracture type. Thirdly, the included studies have different settings for adjustment. Also, we did not have information about recent fracture in the cohorts, and the association between markers and fracture risk may be confounded by a history of prior fracture.

## Conclusions

In conclusion, BTMs hold promise as an independent predictor for fracture. However, before they can be used for this purpose in clinical practice, we need further carefully conducted prospective studies which are analyzing BTMs in a standard manner (such as relative risk per SD increase) for a single fracture type.

## Additional file

Additional file 1: Figure S1. BEGG’s funnel plot of adjusted GR:(A)PINP; (B)CTX. (DOCX 898 kb)

## Abbreviations

BMD: Bone mineral density
BTMs: Bone turnover biomarkers
CI: Confidence interval
CRFs: Clinical risk factors
CTX: C-terminal telopeptide of type I collagen
GR: Gradient of risk
IFCC: International Federation of Clinical Chemistry
IOF: International Osteoporosis Foundation
PINP: Procollagen type I aminoterminal propeptide
SD: Standard deviation
