# Supplementary figures and images for: Reference markers of bone turnover for prediction of fracture: a meta-analysis
Source: J Orthop Surg Res. 2019 Feb 28;14:68. doi: 10.1186/s13018-019-1100-6 (PMC6393999; doi:10.1186/s13018-019-1100-6)

A


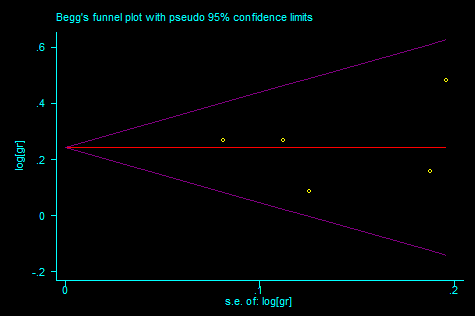


B


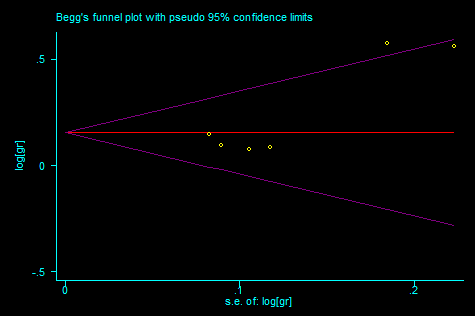


Figure S1. BEGG’s funnel plot of adjusted GR：（A）PINP；（B）CTX

Supplement: Supplementary file 1 — Figure S1. BEGG’s funnel plot of adjusted GR:(A)PINP; (B)CTX. (DOCX 898 kb) [file 13018_2019_1100_MOESM1_ESM.docx]
